# Towards the Development of 3D-Printed Food: A Rheological and Mechanical Approach

**DOI:** 10.3390/foods11091191

**Published:** 2022-04-20

**Authors:** Viridiana Tejada-Ortigoza, Enrique Cuan-Urquizo

**Affiliations:** 1 Tecnologico de Monterrey, Escuela de Ingeniería y Ciencias, Querétaro 76130, Mexico; viri.tejada@tec.mx; 2 Laboratorio Nacional de Manufactura Aditiva y Digital (MADIT), Apodaca 66629, Mexico

**Keywords:** 3D food printing, rheological properties, mechanical properties, additive manufacturing

## Abstract

Additive manufacturing, or 3D printing, has raised interest in many areas, such as the food industry. In food, 3D printing can be used to personalize nutrition and customize the sensorial characteristics of the final product. The rheological properties of the material are the main parameters that impact the 3D-printing process and are crucial to assuring the printability of formulations, although a clear relationship between these properties and printability has not been studied in depth. In addition, an understanding of the mechanical properties of 3D-printed food is crucial for consumer satisfaction, as they are related to the texture of food products. In 3D-printing technologies, each manufacturing parameter has an impact on the resulting mechanical properties; therefore, a thorough characterization of these parameters is necessary prior to the consumption of any 3D-printed food. This review focuses on the rheological and mechanical properties of printed food materials by exploring cutting-edge research working towards developing printed food for personalized nutrition.

## 1. Introduction

Additive manufacturing (AM), commonly known as 3D printing, is a fabrication technology that has raised interest in many areas. It is a manufacturing process that builds up complex solid (or semi-solid) forms by means of a layer-by-layer process. To fuse the layers together, chemical reactions, phase transitions, and some other material properties have been used [1]. Mechanical engineering [2], biotechnology [3], the pharmaceutical industry [4,5], tissue regeneration [6,7], space missions [8], the construction industry [9], and aeronautics [10,11] are only some of the several applications that have been found for this technology; however, it keeps on expanding and maturing into other areas such as food design and development. For the food industry, this increased interest is based partially on advantages such as the simplification of the supply chain and a reduction in storage costs, but mainly on the expansion of the use of existing food materials, customization and design, personalized nutrition, new sensory properties, and other factors [12].

3D food printing has the potential to create geometrically complex products with economic and environmental benefits [13,14,15]. Its greatest advantage lies in the possibility to customize nutrition and sensorial characteristics according to consumers’ preferences and needs [16]. However, to achieve this, a deeper understanding of the rheological properties of the materials used for printing (food inks) and of the mechanical characteristics of the printed materials is needed to assure the feasibility of printing and to achieve novel textures based on the structures printed. When dealing with complex formulations (composed of several interacting food ingredients), rheology may be a technological challenge that can be controlled by changing the chemical composition of the food materials or by the use of gel-type additives [17,18]. Yet, there is still a gap in linking rheological parameters with the feasibility of printing; it is necessary to expand the understanding of those parameters and their usefulness during the printing process. While several determinations have been proposed with the intention of achieving this goal, very few studies have tried to correlate these factors to obtain valuable information assuring the viability of printing with any possible food ink. On the other hand, the characterization of the mechanical properties of 3D-printed food products is crucial. These properties are different from those of the base material prior to printing; given the wide range of manufacturing parameters that can be modified and their impact on the mechanical properties, a structured and standardized characterization is required. Both rheological and mechanical properties must be deeply studied because they directly impact the consumer’s perception.

**Figure 1:**
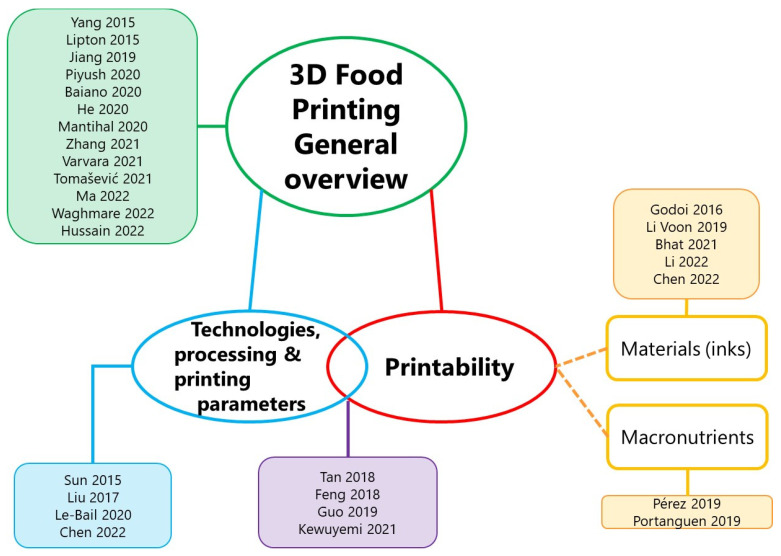
Diagram summarizing 3D food printing reviews published up to date (2015–2022).

Approximately 30 reviews of 3D food printing have been published from 2015 to date, although novel contributions in the literature are narrow in scope. The main topics covered are the printability of food materials, the printability of food macronutrients, the printing parameters, model building and slicing, machines/printers, and future perspectives, among other topics [19,20,21,22,23,24,25,26,27,28,29,30,31,32,33,34,35,36,37,38,39,40,41,42,43,44,45,46,47,48,49]. As a summary of the published literature, a diagram including the previous studies and the topics they focused on is depicted in Figure 1. Although these studies are numerous, in several of them, the information presented is scarcely different. Despite the number of published reviews, an evaluation reveals that none has conducted in-depth research on the rheological properties of the materials to be used for printing food or the mechanical properties of printed inks in order to progress towards the development of 3D-printed food. With this gap in the literature revealed, this review presents a brief overview of the most frequently used techniques to 3D print food, the printing process, and the rheological and mechanical properties of extrusion-based printed food by exploring current cutting-edge research.

## 2. 3D Food Printing Techniques

**Figure 2:**
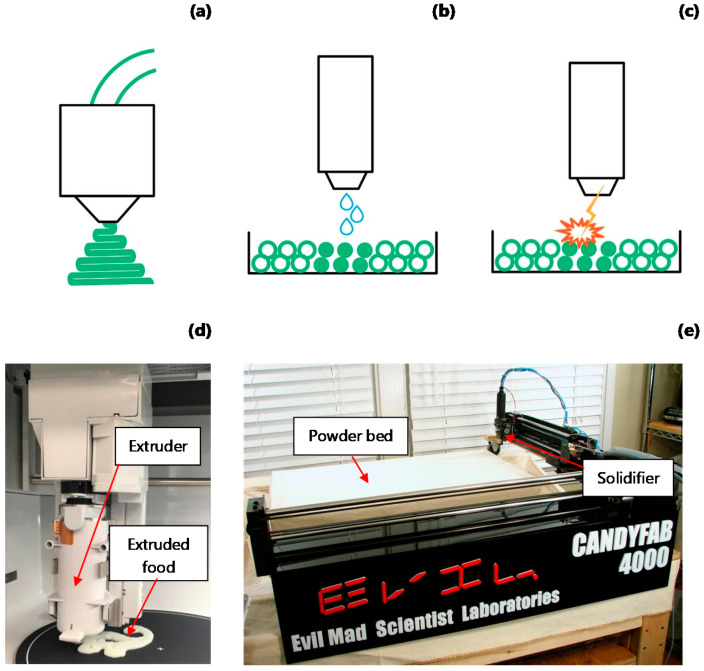
Schematic diagrams and pictures of the most common 3D-printing techniques employed in food processing (**a**) Extrusion-based techniques, (**b**) binder jetting, (**c**) selective laser sintering or melting, (**d**) extrusion-based printer (Foodini from Natural Machines) showing a potato puree dinosaur, and (**e**) powder-based printer with selective melting through hot air (CandyFab 4000, Photo reproduced with permission of Windell H. Oskay, www.evilmadscientist.com (accessed on 17 August 2020), The CandyFab Project, https://candyfab.org/ (accessed on 17 August 2020)).

The process of 3D printing can be achieved by the use of different techniques. Each technique involves different processes depending on the state and form of the raw material used (prior to printing) [28]. In the production of food, the most common techniques are extrusion- and ink-powder-based (such as binder jetting and selective laser sintering) (Figure 2), although the first is the most used and researched. Extrusion-based printing is the most popular process in food production and has been used both for heated- and cold-extruded material. In extrusion-based printers (Figure 2a,d), the extruder is mounted on a three degrees-of-freedom mechanism. This allows the extruder to move in 3D space while staying perpendicular to the base. For hot-extruder-based printing, the food base material, originally in a solid state, is heated up to a semi-molten state before it is extruded. For non-heated materials, the extrusion is highly sensitive to the rheological properties of the material to be extruded. The use of extrusion for printing food has been proved in several types of materials such as cheese [50,51], cereal-based snacks [52], cookie dough [14,53], vegetables [18], meat [54], and others.

In contrast, ink-powder-based 3D-printing technologies consist of a powder material bed that is constantly re-filled. This technique relies on the chemical and physical reaction of the powder to different agents. Binder jetting (Figure 2b) uses a liquid known as a binder to solidify the powder particles, and a mechanism controls the dropping of the binder. Using a similar approach, but with a laser source, selective laser sintering (Figure 2c,e) involves a laser that solidifies by sintering or melting the powder particles, as in the CandyFab^®^ printers. Compared to extrusion-based printing, these techniques are less frequently used for processing complex food materials (with several ingredients interacting), but they have been used with simple food ingredients. For instance, cellulose and xanthan gum were studied to produce cohesive geometric structures for food applications using the binder jetting procedure [12]. A form of selective laser sintering using several food ingredients, such as maltodextrin and starch as structural components and glucose, gluten, maltodextrin, whey protein, and soy protein as binders, was recently patented [55]. Likewise, electrostatic inkjet printing has been used to print chocolate with sufficient accuracy to make complicated, artistic shapes [56].

**Figure 3:**
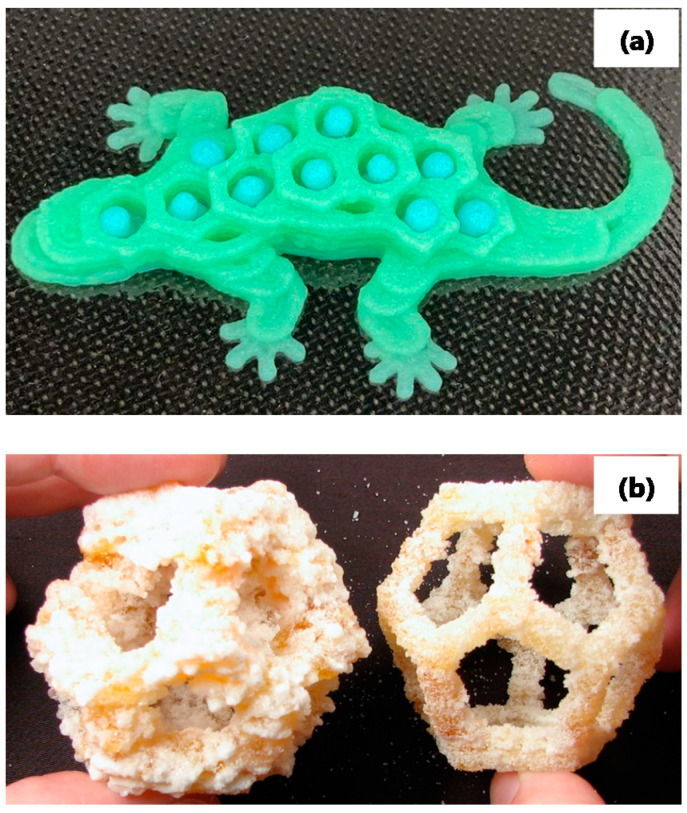
Schematic pictures of (**a**) an extrusion-based food product printed with a Foodini from Natural Machines and (**b**) a powder-based food product printed using a CandyFab 4000 (Photo reproduced with permission of Windell H. Oskay, www.evilmadscientist.com (accessed on 17 August 2020), The CandyFab Project, https://candyfab.org/ (accessed on 17 August 2020)).

The sensory, nutritional, physical, and functional characteristics of the printed products that can be achieved with each technique differ according to the capabilities of the printer. For instance, extrusion-based methods can be more flexible with the use of food ingredients and materials to produce a highly defined printed product, as can be observed in Figure 3. On the other hand, powder-based printers may be less flexible with the use of materials, although complicated 3D structures can still be obtained because the powder bed is used as a support during printing. Food material properties for printing differ widely, even for the same extrusion technique. For instance, during extrusion-based printing, the physical and chemical characteristics of mashed potatoes are different than those of chocolate.

### 2.1. Rheological and Mechanical Properties in the Fabrication Process of 3D Printing Food

**Figure 4:**
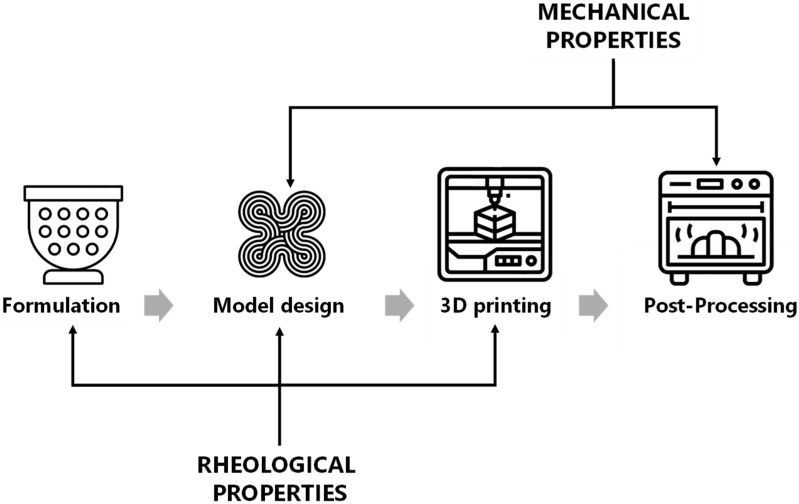
Effects of rheological and mechanical properties through the fabrication procedure of 3D printing food.

The food printing process has four main stages: (a) formulation, (b) model design, (c) 3D printing, and (d) post-processing (Figure 4). *Formulation* is the selection of the materials to be used for the printing process, considering the intrinsic properties of the materials and their printability when mixing all the materials together. It is important to highlight that when formulating complex foods with several ingredients and materials, their compositions interact and can even react. Therefore, formulating complex, nourishing foods has been challenging and scarcely reported on. In this first stage, it is important to consider the rheological properties of the materials because appropriate viscoelastic properties are crucial to allow them to be extruded through the nozzle [35]. Several single materials or very simple food formulations have been successfully printed [17,53,57,58], but printing complex mixtures is more difficult due to the above-mentioned interaction of the materials that leads to changes in their rheological properties [59]. *Model design* refers to the structure arrangement, considering the outside shape and the infill pattern. In this stage, the viscoelasticity of the material is critical to assuring the accuracy and stability of the structure of the printed form. In addition, this stage is strongly correlated to the mechanical properties of the final product based on the infill pattern printed [60,61].

Most 3D-printing processes involve a single processing step to convert the materials into a final product; however, this is currently complicated when applied to food and when integrated into a kitchen or industrial-scale production. When dealing with food, a final step, or *post-processing*, such as baking, frying, or drying, may be needed to maintain the shape, assure microbiological safety, prolong shelf-life, or make it sensorily acceptable to consumers. Post-processing also has an important effect on the mechanical properties of the 3D-printed material [35]. The following sections review both rheological and mechanical properties as important parameters to be considered during the extrusion-based process. It is important to highlight that the printing parameters (set in the 3D printer] are outside the scope of this work, but they have been studied in depth [22]. Printing parameters depend mainly on the capacity of the printer used and may also vary with the food ink.

### 2.2. The Effect of Rheological and Mechanical Properties on Texture

Regarding food, texture and mouthfeel can be crucial for consumers’ preferences and acceptability [62]. According to the International Organization for Standardization, texture is defined as “all the mechanical, geometrical and surface attributes of a product perceptible by means of mechanical, tactile and, where appropriate, visual and auditory receptors” [49,62,63]. As noted in Figure 4, when 3D printing food, rheological and mechanical properties have an important effect on texture. Rheological properties and their impact on texture are evaluated using the printable materials, either before printing or once printed. In the case of mechanical properties, the approach can be based on the printable materials but may also be based on the printed and post-processed (e.g., fried, dried, or cooked) materials.

For instance, some works have evaluated the application of 3D printing to developing food products for people with dysphagia, a common problem in elderly populations [64,65,66]. Kouzani et al. [66] have printed tuna fish, pumpkin, and beetroot purees for people with swallowing difficulties; they printed them with a tuna fish shape design. In order to be appealing not only to these but to all consumers, 3D-printed food must be consistent in terms of the sensory experience as well as visually attractive. Moreover, it is important to highlight that nourishing formulations also have to be also achieved [64]. To assure this, the printability of the materials, including their design and suitability while printing, is one of the most important parameters for the successful development of such products (Section 3).

The precise placement of texturing elements in the food and the design of complex internal structures result in an important effect on the mechanical properties. For the development of these inner structures and their mechanical evaluation, post-processing is commonly performed. The manipulation of texture may lead to the creation of healthier products with reduced salt, sugar, or fat content [49]. A deeper assessment regarding the texture profile analysis (TPA) and the implications on how this test has performed in 3D-printed materials is covered in Section 4.

## 3. Rheological Properties of 3D-Printed Food

The main parameter affecting extrusion-based 3D-printing processes is the rheology of the food ink. A printable material needs rheological properties that allow extrusion through the nozzle and a fast stabilization once the material has been deposited to guarantee the fidelity of the shape. In addition, the printed object should not collapse during printing and/or post-treatment [35]. During the extrusion process, a mechanical load is required, and its magnitude is mainly related to the rheological properties of the material but also to the geometry of the nozzle [67,68]. Pseudoplastic materials are ideal for 3D-printing extrusion processes because they have the ability to flow through narrow nozzles at high speeds. Once the stress applied exceeds the yield stress inside the extruder, the material must exhibit the capacity to hold its structure after the extrusion process, conforming to the 3D model (print fidelity). In addition, the extruded material must be capable of adhering to the previously deposited layers [18,69,70]. As material flows out of the moving nozzle during its deposition, kinetic energy decreases because of viscoelastic effects (conversion to elastic energy and/or heat dissipation) until the shear stresses coexisting between the material at the leading-edge fall below the yield stress. At this point, the flow stops and leads to defined edges of the deposited food materials [71]. In this regard, it was necessary to define a concept that incorporates the rheological properties of the materials to be printed, and the term *printability* emerged.

Printability is defined by two main material characteristics: (i) its intrinsic properties that facilitate handling and deposition by the printer (flowability through the nozzle), and (ii) its capacity to hold the structure and dimensional stability, either as the final step or until post-processing [21]. The intrinsic properties of the food material are closely related to its macronutrient content, determining the rheological and physicochemical properties [26]. Accordingly, some authors have made a general classification of food materials as printable or non-printable [72,73,74], although classifying and determining the printable nature of complex materials such as food may be a difficult task because several parameters such as temperature, food components, and additives, among others, may affect it. In addition, the thixotropic behavior of the materials must also be considered [75].

Printable materials, such as some confectionary products and hydrogels, are easy to extrude and their shape is well maintained once deposited. In chocolate, for example, cocoa butter (fat) is the main ingredient responsible for the structural behavior [26]. Gel-type materials, such as hydrogels and some proteins, have been widely used for 3D-printing purposes due to their capability to hold large amounts of water through physical and chemical mechanisms that provide them with strong structural characteristics [76,77]. Some authors have reported that a printable material has enough flow ability to be extruded through the nozzle without any additional materials that enhance this property [74].

Non-printable materials are hard to extrude because they can be too thick to pass through the nozzle, exceeding the force applied by the printer to make it flow or, even because they can lose consistency once printed even if they make it through the nozzle. However, their high nutritional content makes them of interest for printing. Meat, for instance, is a fibrous material that requires the modification of its rheological and mechanical properties to make it an extrudable, paste-like material. Flow enhancers are commonly used to improve the printability of these materials [74]. Further information regarding materials such as animal products that have been developed using 3D printing can be found in [41]. Commonly, powder-based materials and water are used to formulate a printable mixture. Powder characteristics such as volume, particle size, and swelling may impact the rheological properties of the material. In addition, water content is the main factor affecting the printability of food materials; thus, moisture control methods may ease the achievement of convenient rheological properties for printing through extrusion processes [18].

### Rheological Parameters Used to Characterize Food Ink Materials

Some rheological parameters have been used to understand the 3D-printing process during extrusion through the nozzle and once the material has been printed; however, more research is needed in this area. As a starting point, Table 1 presents some of these parameters and the information for 3D-printing behavior that has been obtained from them. Flow behavior index (*n*), consistency index (K), and viscosity are parameters that have been used for 3D printing, although these are less commonly reported in the literature. These parameters have been related to the shear-thinning behavior of the materials and the ease of being extruded through a nozzle. Based on these parameters, several authors have reported the rheological behavior of the studied materials and made conclusions about their specific characteristics for printing. The viscosity of printing materials should be low enough so that they can be extruded through a nozzle but high enough to maintain the desired shape [78].

By definition, yield stress is the minimum shear stress that must be applied to the material to initiate flow [79]. It has been stated that the yield stress can be used to evaluate the extrudability of food inks because it is associated with the mechanical strength of the material [53,80]. However, this parameter by itself does not guarantee the printability of food materials [80]. Some other parameters commonly reported are the storage (G’) and loss modulus (G’’) of food inks, which provide information about the solid-like or viscous-like behavior of the material. Altogether, a high value of yield stress and a high value of elastic modulus minimizes the deformation of the food ink once deposited and avoids the collapsing of the 3D structure [81].

Even though these parameters have been useful for the characterization of food materials, there is still a need to broaden the methodologies for characterizing the technological feasibility to print food. Table 2 shows the rheological parameters commonly reported when 3D printing food, as stated. It can be observed that several materials with different characteristics and compositions have been studied: hydrocolloids, fibers, carbohydrates, fats, etc. Because of these different characteristics, extrinsic and intrinsic, the rheometer settings used are obviously different, mainly in the use of parallel plain or serrated plates and in the varying sizes of these plates. In addition, a wide range of temperatures has been tested depending on the material. Table 2 aims to show the wide difference among yield stress, K, *n*, G’, G’’, and tan δ values that have been obtained by the authors that studied these materials. Moreover, the published works do not always report the same parameters, and comparisons are virtually impossible and, in many cases, unfair due to the different settings and materials used. The complexity of food inks lies in the broad differences in their rheological behavior that depend on food composition, chemical interactions, environmental conditions, etc. This does not allow for the establishment of rheological value ranges where a food is printable, because all food inks must be treated differently, and some other parameters such as printability, as opposed to only rheological parameters, must be used. For instance, chocolate behaves differently when compared to a vegetable-hydrocolloid mixture. However, in general, it is also important to note that G’’ values are lower than G’ values in all presented cases, meaning that a viscoelastic behavior should be exhibited by the material and that a solid-like behavior should be prevalent during printing.

Some works have aimed to correlate printability with rheological properties, although wider conclusions are still needed [67,73]. For instance, a recent work quantitatively related the rheological properties of food pastes to printing stability and extrusion force [67]. These authors related the stress at collapse with the flow stress, zero shear viscosity, and storage modulus. The stress at collapse was obtained by dividing the total sample weight at the collapse height by the bottom surface area of the printed figure. The authors reported a linear correlation between the evaluated rheological properties (flow stress and zero shear viscosity) and the printing stability. In addition, they proposed a decision algorithm to develop aqueous food recipes with the desired printability based on flow stress obtained by shear rheology. While this work is possibly one of the first attempts to establish a quantitative correlation of printability with rheology, stress at collapse may not be the best parameter for this comparison. This is because structural failure during the printing process is closely related to the shape and mechanical performance of the type of product printed and the distribution of infill material within it [95,96,97].

**Figure 5:**
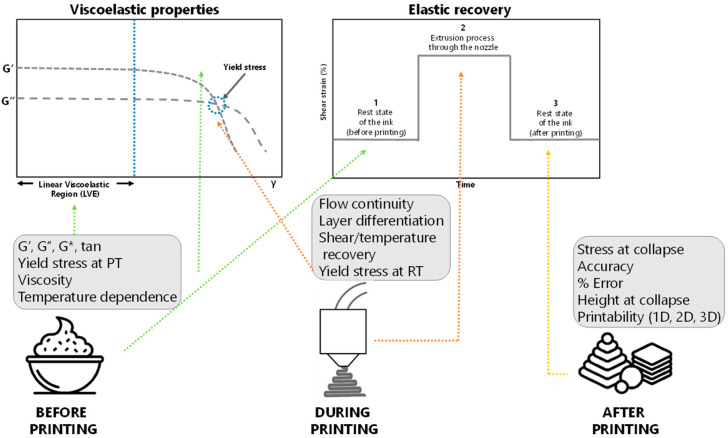
Evaluations used for the characterization of the rheological parameters and printability of food materials before, during, and after the printing process. PT—printing temperature, RT—room temperature.

Moreover, it is crucial to differentiate among the methodologies or techniques that are used to characterize printability of the materials before, during, and after printing (Figure 5). The characterization of the shear-thinning behavior (the flow sweep test), the viscoelastic properties (the amplitude sweep test), the elastic recovery (the three interval thixotropy test), and the temperature dependence of viscoelastic properties (the temperature ramp) is crucial for assessing the printability of materials [81], although some other less common methods have also been reported. For instance, viscoelastic properties and viscosity have been mainly used to characterize food ink materials before printing. G’ and G’’ are obtained from the linear viscoelastic region, and some authors have reported that the yield stress should be obtained at the printing temperature to estimate the information regarding the stress needed to print [84]. Yield stress must also be determined at room temperature to obtain information about the mechanical strength of the material during the printing process [84]. In addition, some other methodologies, such as flow continuity, layer differentiation, shear recovery, and temperature recovery, have been proposed for this stage. Finally, once the material is printed and deposited, stress at collapse, accuracy, error percentage compared to an ideal printing area, height at collapse, and printability in 1D, 2D, and 3D have been used for the characterization of the printed figures [67,73,84].

Rheological properties must be studied to establish parameters that assure that the printing of a mixture is technologically feasible, meaning that it passes through the nozzle and keeps its form and internal structure once extruded or post-processed. This is needed if the 3D printing of food is to move towards healthy formulations for personalized nutrition, which generally cannot currently be printed or extruded because of the complexity of the mixture and the interaction of the ingredients.

## 4. Characterization of the Mechanical Properties of 3D-Printed Food

While the term used by the food engineering community is texture analysis, for mechanical and materials scientists, it is essentially the mechanical characterization of material properties. The so-called *texturometer* is known as a universal testing machine for mechanics and materialists. Therefore, many of the concepts and contributions derived from the study of the mechanical properties of 3D-printed parts can be applied when dealing with edible printed material. This was recently discussed by Peleg [98]. Several parameters, although not all, that could affect the mechanical properties of food (i.e., texture, snap, chewiness, gumminess, etc.) can be taken from the work done on non-edible materials and are related to the stiffness, strength, deformation mechanisms, hardness of the materials, and 3D-printed structures. These parameters were summarized in a recent work [97] that covers a review of the literature on the mechanical characterization of 3D-printed parts fabricated via extrusion.

**Figure 6:**
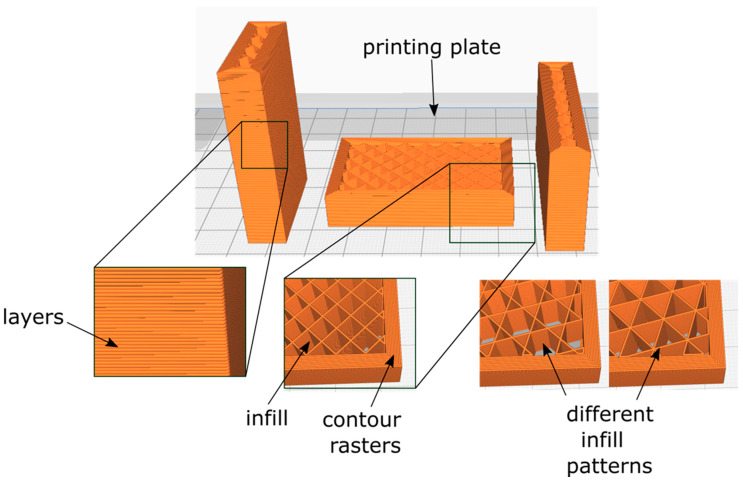
Printing parameters that have an influence on their mechanical properties. Diagrams obtained from CURA^®^.

Numerous parameters have an effect on the resulting mechanical properties of 3D-printed samples: (i) building orientation, (ii) infill structure, and (iii) infill density, among others [97]. These are illustrated in Figure 6, where a computationally modeled sample shows three different orientation possibilities. (i) *Building orientation* is significant, as 3D-printed products are known to be anisotropic, meaning that their mechanical properties depend on direction [99]. (ii)–(iii) *Infill* is the lattice or tessellated arrangement used to fill 3D-printed products.

When dealing with food, the concept of TPA is the most used for characterizing the mechanical properties of food products (material). However, when characterizing 3D-printed food using TPA, one can obtain misleading results because the measured properties may be related to specific manufacturing parameters or to sample dimensions and shapes. Results obtained from these testing procedures are specific; changes in sample dimensions and/or shapes may result in different measured properties. Proper sample design is needed so that measured properties are universal. For instance, characterization of the mechanical properties of the infill would be more useful if the samples were printed without contour rasters. When contour rasters are aligned to the principal axes of the sample, they are more likely to withstand most of the load, leading to an insignificant contribution by the infill. Most infill patterns are known to have in-plane anisotropy. A complete understanding of their mechanical properties demands samples with different infill structure orientations to characterize their dependency on the loading direction.

TPA analyses have been performed to characterize both edible printing material (mixtures and slurries prior to printing) and printed food products. This analysis has been used mainly for compression [93] and bending (usually called *cutting*). Characterizing the material properties prior to printing is crucial for evaluating the printability of products and the rheological and viscosity measurements, as discussed in Section 3. Several works that deal with a variety of printing materials are available, such as studies of egg white protein mixtures [68], dairy protein mixtures [94], cheese [51], methylcellulose and gum mixtures [13], lemon [58], orange [100], and potato puree [101].

In this section, the review of the literature focuses on work wherein the mechanical properties were characterized using 3D-printed samples. However, one needs to be aware that when characterizing any 3D-printed product via TPA, the results are not associated purely with the material that is being used but also with the combination of materials and the fabrication parameters, such as post-processing (Figure 4 and Figure 6). Both, keeping the same base material while changing printing parameters and changing the base material while keeping the printing parameters constant lead to changes in mechanical properties. This makes 3D printing a versatile fabrication technique for the production of food with customized properties.

### 4.1. Effect of Infill Density

Among 3D-printing techniques, extrusion-based techniques are more frequently encountered, both with hot-extruder and non-heated processes. Extrusion machines are based on a Cartesian mechanism that uses three servomotors to control the position of the extruder in the three principal axes. This allows building parts (food) from the stack of extruded material. Extruded material is deposited in “2D” layers conformed by contour rasters and the infill. It has been shown that the infill affects the mechanical properties of the printed part [60]. The important thing in the field of food printing lies in the different sensorial perceptions of consumers provided by the variation in the internal structure patterns [91,102]. This was studied in [52], where cereal-based products were printed and compressed. Products made from chocolate extrusion were printed with different infill patterns, and their *snap* [buckling] properties were analyzed [103].

**Figure 7:**
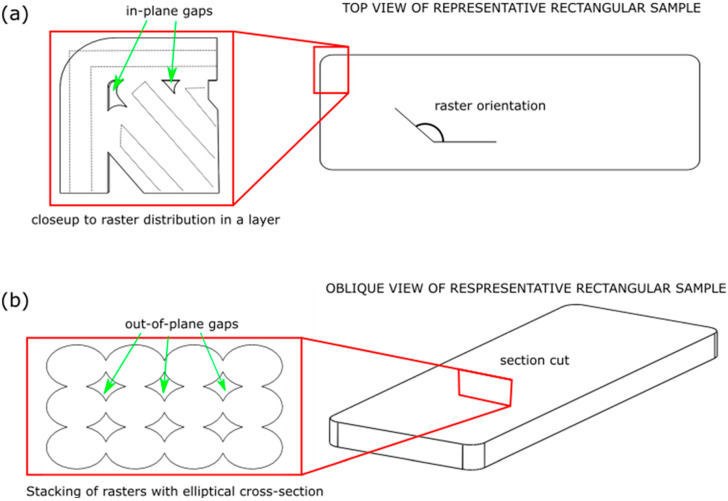
Inevitable porosity in parts fabricated using extrusion-based 3D-printing processes: (**a**) in-plane and (**b**) out-of-plane gaps.

When mechanical properties show negligible differences while testing different material compositions, an alternative could be changing the printing parameters [104]. This can be achieved by manipulating the infill structure and density. The effects of infill density and topology on the texture of 3D-printed mashed potato have been determined by [105]. Minimal differences were observed when modifying the infill pattern type; this was attributed to the compressive testing direction of the samples. In this regard, consumer perception can be modified by changing printing parameters, as studied by [57], where chocolate samples were fabricated with different infill densities and compared against cast samples. The 100% filled samples resulted in lower forces at break than the casted samples (a difference of about 10 N). This demonstrates that, even when the infill percentage is set to its maximum, the extrusion raster direction and unavoidable porosity (Figure 7) affect the mechanical properties. Subsequently, reductions in the forces at break in the range of 2.6 to 1.6 times were obtained when reducing the infill percentage from 100% to 50% and 25%, respectively.

Other authors presented a detailed study on mashed potato samples, where three different infill densities were tested, along with variations in contour rasters and infill patterns (Figure 7) [105]. Cylindrical samples were subjected to compression tests; as expected, a higher number of contour rasters or higher infill densities resulted in a higher measured Young’s modulus. 3D-printed samples were also compared with molded samples; even those that were printed with a 100% infill density resulted in lower properties than the molded samples. Differences obtained were in the range of 50 KPa. As mentioned, due to the nature of the process, even parts fabricated with a 100% infill density resulted in inevitable porosity. This porosity results mainly from two types of gaps: (i) in-plane gaps generated due to the impossibility of fully filling the layer (Figure 7a) and (ii) out-of-plane gaps resulting from the stacking of rasters with circular or elliptical cross-sections (Figure 7b) [106]. Additionally, differences between fully-dense printed parts and injected molded ones can be attributed to their anisotropy, inherent in parts fabricated with extrusion-based processes [99].

Finally, some authors have tested different compositions of potato by-products and yam on disc-like 3D-printed samples [107]. Samples 3D-printed with different infill densities (20%, 50%, and 80%) were subjected to three-point bending, and the PF was reported. Differences in the measured peak force were more significant for those measured at different values of infill densities (incrementing roughly 30 N for every increment in density of 30%) than the difference between different compositions (statistically insignificant in most of the cases).

### 4.2. Effect of Building Orientation

Yang et al. [58] evaluated the peak force, energy, and springiness of lemon juice gels containing potato starch (10–20 g/100 g) while keeping the printing parameters constant. As all samples were printed with the stacking direction parallel to the principal axis, differences in peak force measurements are only attributed to the composition of the base material. When a product has been 3D-printed by stacking the layers in the axis normal to the printing plate and is loaded in this same direction, the mechanical properties are governed by the local deformation at the bonding between layers.

Additionally, the anisotropy inherent in structures fabricated with extrusion-based techniques is still an open question for 3D-printed food. In order to fully characterize the mechanical properties, i.e., hardness, gumminess, strength, elasticity, and texture, testing should be performed along different directions of the printed samples [108]. A possible attempt to achieve isotropy, at least in the printing plate plane, could be achieved by making use of cellular materials [109]. Hexagonal honeycombs are known to have in-plane isotropy under specific conditions [110]. Some authors have printed pectin-based food simulants in hexagonal honeycombs structures, predicting the mechanical properties by an analytical model and finite element modeling [109]. In this work, the authors compared the structure features of the printed objects to those estimated by computational mechanical simulations and analytical models [110]. The effective Young’s modulus of the pectin-based samples showed a non-linear relationship with the geometrical parameters that defined the honeycomb; this is in agreement with what was predicted by [110].

When testing a 3D-printed air-fried potato snack along the stacking direction, the more the surface area of each layer was in contact with the previously extruded layer, the higher the hardness (peak force) measured in the samples [111]. Even with variations in infill pattern topology while keeping the infill density the same, the variations in peak force were minimal [111]. Changing the topology of the infill while keeping the density the same, the area of a single layer was almost the same as the previously deposited one. The influence of infill density may be more evident in the in-plane (building plane) properties [57,107].

### 4.3. Perspectives on the Mechanical Properties of 3D-Printed Food

The ideas exposed here allow the interested reader to form an idea of the vast parameters that can be modified to adjust the resulting mechanical properties. This is one of the advantages that characterize additive-manufactured parts (not only food). The mechanical properties that can be achieved depend upon the selection of the parameters. For example, two samples made of the same base material but tested or fabricated along direct directions result in different properties. Another example that may be encountered is having the same base material and different outer shapes and dimensions, leading to different mechanical properties. Hence, important insights need to be generated by this review that are applicable to 3D-printed food. Standards for the characterization of the mechanical properties of 3D printing food are not only a necessity but also an urgency. These standards should include not only testing setup but also manufacturing parameters.

Each work that publishes findings on the characterization of mechanical properties uses a different material (food) composition, fabricates samples with different shapes and dimensions, and tests under different conditions. Hence, a direct comparison among the data available in the literature yields inequitable results. Despite the fact that comparisons of the mechanical properties reported in different works are unfair, here Table 3 presents a summary of some of the recent works that include texture analyses. Note that a column that mentions the parameters varied and the shape of the samples is included. This is important for future research, as the properties reported are particular to these works, and readers must not take them as generalized properties, even if they use the same base materials and nutrients.

## 5. Future Insights

Once rheology and mechanical properties are controlled, the post-processing of printed food must be studied in depth to finish the process, even though it is not required for all types of printed food. Post-processing is directly impacted by rheology because the printed structure must maintain its shape until the end of the process, but it also impacts the final texture of the food sample. In this regard, a multi-material printing process with simultaneous infrared cooking was recently reported. The authors integrated an infrared lamp heating mechanism into the printer for the precise control of the heat delivery to the food material. In their work, they designed the printer and tested several food materials with different cooking times, with successful results for the simultaneous printing–cooking process [114]. While this is an innovative system for 3D printing and cooking food, several authors have studied other, different post-processing techniques that are not simultaneous, such as baking [52,80,115], air-frying [107], and drying [86], among others. The importance of post-processing in 3D food printing has been studied in depth by [35].

In addition, the concept of 4D printing has also emerged. Herein, the addition of the “space–time axis” is included. Shape, texture, taste, nutrient composition, etc., may be changed by stimulants such as water, heat, light, or pH [116]. Starch gels, hydrogel systems, and soy protein isolate, to name a few, have been used for 4D printing and to induce changes; in some cases, post-processing is needed. For instance, starch-based purees from purple sweet potatoes have spontaneously changed their shape after using microwave dehydration [117]. An induction of color change in mashed potato/purple sweet puree potato samples was performed after changing the pH [118], similar to [119]. Although this technology is still exploratory, Teng et al. [116] highlighted the importance of a synergy between the internal structure that can be designed and the stimulation of printed food. Additionally, new materials must emerge to create new printing conditions and innovative property changes [116].

Finally, future insights must be aligned with the acceptability of 3D-printed food products to consumers, along with the nutritional benefits they can obtain. There is still a gap to be filled in this sense because only a few works have focused on this [120,121,122]. The vast majority started using 3D food printing as a novel fabrication technique with a high degree of innovation while neglecting crucial aspects important to the consumer. It has been reported that consumers tend to reject novel technologies if they associate them with health risks or if they believe that harmful by-products might be produced. In contrast, health benefits and improved sensorial properties enhance consumer acceptance [121]. In this regard, personalized nutrition is one of the most important applications in the future of 3D printing. Customized flavors, nutritional composition, shapes, textures, etc., may be achievable using this technology. However, consumers have suggested that food inks must be available in the market so that the printing process is ready-made, making it more convenient. This also assures that technical knowledge of the sort presented in this work (rheology) is not required [122]. The food ink market is a huge opportunity for the food industry that opens up the possibility of broadening the materials that can be used.

## 6. Conclusions

Food 3D printing has recently gained attention due to several advantages, such as the expansion of the use of existing food materials, customization and design, and personalized nutrition, among other factors. This work reviewed the main 3D-printing techniques for processing foods, with the extrusion-based method the most currently used. Rheological and mechanical properties were reviewed in depth because they are some of the main characteristics that impact the feasibility of the technology and the 3D food printing–consumer relationship. Because one of the main advantages of the technology is the customization of nutrition, more research needs to be done related to complex and healthy formulations to satisfy the nutritional needs of targeted groups. However, providing healthy formulations is not enough if those formulations cannot be printed or extruded; thus, the study of rheological properties must be explored in depth to determine parameters that assure that the printing of a certain mixture is technologically feasible. Wide differences among the rheological properties of food inks have been reported in the literature, so comparisons and references are virtually impossible to set. However, the relationship of these rheological parameters to printability must be established and studied in depth. The methodologies that have been used to obtain information about the printability of the materials by using rheology before, during, and after the printing process were discussed. The lack of studies that relate rheological parameters to printability and the need to model and generalize printing parameters using the rheological properties of the materials are some of the practical and theoretical implications derived from this research.

Finally, given the wide range of possibilities that 3D-printing technology offers in terms of shape design freedom, current and future efforts should be focused on standardizing the characterization procedures. 3D-printed food products, while being made of the same (or similar) base materials, can result in different mechanical properties if the macro shapes or the loading conditions differ. One must understand which properties should be universal (those related to the material) and which are modifiable given the manufacturing parameters employed for fabrication. 3D-printing technologies are employed under the control of several parameters, and each, in turn, has an impact on the resulting mechanical properties. Here the review covered the most influential parameters, i.e., printing orientation, infill density, and topology. Material distribution within the 3D-printed product, the topology, and the shapes of the printed products are relevant because they affect the mechanical properties of the final product. These are crucial when dealing with the consumer’s acceptance or when trying to target a specific population (e.g., elderly people with swallowing problems). The main limitation of the present work is that, although a general overview and explanations regarding the rheological and mechanical properties of 3D-printed materials are presented, there is a lack of standardization in the evaluation of such properties. This makes comparisons among different works inequitable, so the need for standards for the characterization of the above-mentioned properties is not only a necessity but also an urgency for upcoming studies.

Future work should include a characterization of the effect of these parameters under other loading scenarios, e.g., tension (stretch) and torsion (twist). More research will continue to emerge towards the evolution and presence of 3D printing food in society; however, more effort is required to set healthier formulations with sensorial acceptance.

## Figures and Tables

**Table 1:** Rheological parameters used for the prediction of extrusion behavior and printability.

Parameter	Definition	Correlation with 3D Printing
Flow behavior index, *n*	Parameters of the power law applied to fluids	Low values indicate high shear-thinning properties that can be easily extruded out of a nozzle when increasing shear stress is applied.
Consistency index, K		High values are associated with materials not easily extruded from the nozzle.
Viscosity	A measure of a fluid’s resistance to flow. It is also a relation between shear stress and shear strain	High-viscosity materials easily stick on the extruder walls and block the nozzle output. Thus, an inaccurate production of the final shape of the product may be obtained.
Yield stress (τ_o_)	The minimum shear stress that must be applied to the material to initiate flow	- May indicate self-support. - Above this value, the structure of the material breaks and flows because the internal structure cannot hold the pressure and store the energy.
Storage modulus (G’)	Defines the solid-like behavior and reflects the mechanical strength of materials	In combination with the yield stress, this has been used to predict the shape retention of a printed material and a good resolution (printing fidelity).
Loss modulus (G’’)	The viscous response of the material	G’ and G’’ values indicate the ability of the matrix to support itself once printed. These parameters give valuable information about structure because a strong frequency dependence might indicate a material structure that behaves like a solid at higher frequencies and like a liquid at lower frequencies.
tan (δ) = G’’/G’	-	High values indicate a fluid-like behavior, and low values a solid-like behavior. So, if G’ ≥ G’’, then a resistance against collapse and a better holding of shape after printing is observed.
Shear modulus	The ratio of shear stress to shear strain in a body	- Has been used to predict the shape deformation. - Predicts and quantifies the deformation behavior after the printing process.

References: [18,26,45,69,78,82,83].

**Table 2:** Rheological parameters reported for varied materials used for 3D printing.

Material	Rheometer Settings	Yield Stress (Pa)	K (Pa s^n^)	*n* (Dimensionless)	G’ (Pa)	G’’ (Pa)	G*	Tan δ	Reference
Carrageenan-xanthan-starch	Parallel plates, diameter of 40 mm, gap of 0.2 mm, 35–45 °C,	12–550	7–24	0.48–0.36	50–9000	40–1000	60–9000	-	[84]
Mixtures of high and low gluten wheat flour, sugar, butter, water, and potato granules	Parallel plates, diameter of 25 mm, gap of 1 mm, 20–35 °C	-	-	-	10,000–180,000	2000–78,000	-	0.35–0.53	[85]
Starch, cellulose nanofiber, milk powder, oat, and faba bean protein-based materials and their mixtures	Stainless steel parallel plates, diameter of 20 mm, gap of 1 mm, 22 °C	5–61	-	-	260–1900	43–320	-	9.5–10.6	[86]
Agar- and Konjac-based edible gels	Parallel plates of 25 mm, gap of 0.8–1 mm, 25 °C	-	-	-	100–800	10–60	-	-	[87]
κ-carrageenan hydrogels	Parallel plates of 25 mm, gap of 1 mm, 25 °C	-	-	-	-	-	-	-	[88]
Cheese	Parallel plates of 20 mm, 25 °C	-	-	-	-	-	32,000–66,000	0.29–0.35	[51]
Potato puree	Parallel plates of 25 mm, gap of 1 mm, 25 °C	-	19–612	0.12–0.51	1000–9000	100–1700	-	-	[89]
Egg yolk	Parallel plates of 60 mm, gap of 1 mm, 25 °C	-	-	-	500–1000	250–800	-		[78]
Cheese	Serrated parallel plates of 25 mm, 20.5 °C	-	-	-	25,000–49,000	-	-	0.25–0.31	[90]
Peanut butter, rice-starch gel, and cream cheese	Serrated and flat parallel plates of 25 mm, gap of 1 mm, 22 °C	7–47	-	-	-	-	2200–67,000	-	[71]
Vegetable and xanthan gum (30%)	Sandblasted parallel plates of 25 mm, gap of 1 mm, 25 °C	-	-	-	7000–9000	1500–1800	-	-	[18]
Lemon juice gel	Parallel plates of 20 mm, 25 °C	-	-	-	500–5000	150–1800	-	-	[91]
Cookie dough	Serrated parallel plates of 40 mm, gap of 2 mm, 25 °C	7–285	-	-	-	-	-	-	[80]
Mashed potato	Parallel plates, diameter of 20 mm, gap of 2 mm, 25 °C	195–370	-	-	1200–7500	300–2500	-	0.18–0.39	[83]
Fish surimi gel	Parallel plates of 20 mm, gap of 2 mm, 25 °C	-	-	-	10,000–250,000	4000–60,000	-	0.2–0.5	[92]
Vegepate and tomato puree	Serrated parallel plates of 25 mm, 25 °C	-	-	-	4000–15,000	1000–3000	-	-	[67]
Brown rice	Parallel plates of 20 mm, gap of 2 mm, 25 °C	800–2100	-	-	20,000–30,000	3000–4000	-	-	[93]
Milk protein concentrate and whey protein isolate mixtures	Parallel plates of 35 mm, gap of 1 mm, 25 °C	-	-	-	20,000–70,000	1000–30,000	-	-	[94]

**Table 3:** Texture analyses performed in 3D food printed materials.

Base Materials	3D-Printing Parameter Studied	Properties Characterized	Shape of the Sample	Type of Test	Maximum and Minimum Values Reported	Reference
Protein bar with chocolate	Infill density, infill topology	ST, H, C, Ch	Square/prism	Compression	ST: 0.7–2 MPa, H: 200–400 N, C: 0.04–0.07, Ch: 0.5–2.3 N	[112]
Lemon juice gel	Nozzle diameter	H, SP, C, Gu	Cylindrical	Compression	H: 1.48–3.98 N, C: 0.65–0.94, SP: 0.85–0.94, Gu: 9.98–379.74	[58]
Protein, starch, and fiber	Air pressure in extrusion	H	Square plate with lattice	Cutting	H: 2.9–59.8 N	[86]
Various gums	-	H, SP, C	Square/prism, cylindrical, and triangular	Compression	H: 1.72–2.94 N, SP: 0.75–0.9, C: 0.7–0.8	[104]
Cereal based	-	H	Cylindrical with inner square structure	Compression	H: 20–52 N	[113]
Mashed potato	Infill density, infill topology, perimeters	H, Gu, ST	Cylindrical with infill patterns	Compression	H: 1.16–3.92, Gu: 30–150, ST: 0.0004–0.04 MPa	[105]
Chocolate	Infill density	H	Prismatic bars	Compression	H: 20–71 N	[57]
Cereal-based	Infill density, layer height	H	Cylindrical with inner square structure	Compression	H: 10–70 N	[52]

ST: stiffness, H: hardness, C: cohesiveness, Ch: chewiness, SP: springiness, Gu: gumminess.

## Data Availability

Not applicable.
